# A data-driven approach for the partial reconstruction of individual human molar teeth using generative deep learning

**DOI:** 10.3389/frai.2024.1339193

**Published:** 2024-04-16

**Authors:** Alexander Broll, Martin Rosentritt, Thomas Schlegl, Markus Goldhacker

**Affiliations:** ^1^Department of Prosthetic Dentistry, University Hospital Regensburg, Regensburg, Germany; ^2^Faculty of Mechanical Engineering, Ostbayerische Technische Hochschule Regensburg, Regensburg, Germany

**Keywords:** inlay restoration, dental prosthesis design, StyleGAN, machine learning, digital dentistry

## Abstract

**Background and objective:**

Due to the high prevalence of dental caries, fixed dental restorations are regularly required to restore compromised teeth or replace missing teeth while retaining function and aesthetic appearance. The fabrication of dental restorations, however, remains challenging due to the complexity of the human masticatory system as well as the unique morphology of each individual dentition. Adaptation and reworking are frequently required during the insertion of fixed dental prostheses (FDPs), which increase cost and treatment time. This article proposes a data-driven approach for the partial reconstruction of occlusal surfaces based on a data set that comprises 92 3D mesh files of full dental crown restorations.

**Methods:**

A Generative Adversarial Network (GAN) is considered for the given task in view of its ability to represent extensive data sets in an unsupervised manner with a wide variety of applications. Having demonstrated good capabilities in terms of image quality and training stability, StyleGAN-2 has been chosen as the main network for generating the occlusal surfaces. A 2D projection method is proposed in order to generate 2D representations of the provided 3D tooth data set for integration with the StyleGAN architecture. The reconstruction capabilities of the trained network are demonstrated by means of 4 common inlay types using a Bayesian Image Reconstruction method. This involves pre-processing the data in order to extract the necessary information of the tooth preparations required for the used method as well as the modification of the initial reconstruction loss.

**Results:**

The reconstruction process yields satisfactory visual and quantitative results for all preparations with a root mean square error (RMSE) ranging from 0.02 mm to 0.18 mm. When compared against a clinical procedure for CAD inlay fabrication, the group of dentists preferred the GAN-based restorations for 3 of the total 4 inlay geometries.

**Conclusions:**

This article shows the effectiveness of the StyleGAN architecture with a downstream optimization process for the reconstruction of 4 different inlay geometries. The independence of the reconstruction process and the initial training of the GAN enables the application of the method for arbitrary inlay geometries without time-consuming retraining of the GAN.

## 1 Introduction

Between 1990 and 2017, oral and headache disorders held the top two positions in the Global Burden of Disease Study ranking for level 3 causes (James et al., 2018). Medical causes of diseases can be categorized into different levels based on their complexity and specificity. Dental caries, commonly known as tooth decay, is the principal cause of dental treatment for oral disorders and its high prevalence is associated with the necessity of the fabrication and insertion of FDPs. FDPs include partial crowns, crowns, and multi-unit FDPs, which are typically made from ceramics, alloys or resin-based materials. These restorations play a crucial role in maintaining the correct function of the masticatory system, which encompasses both static contacts in static occlusion and dynamic contacts during excursive movements of the lower jaw with teeth in contact. Static occlusion denotes the point of contact between teeth when the jaw is in a closed and motionless position. To produce well-fitting restorations that accurately reproduce static contacts between the teeth of the upper and lower jaw, 3D scans and CAD/CAM systems are commonly utilized. However, simulating dynamic occlusion in the dental laboratory and transferring the patient's individual movements into an ideal geometry of the FDP is a complex task.

Especially for smaller restorations, the design of FDPs is currently based on a functional analysis of the patient, which allows the parametrization of mechanical or digital articulators to verify that the produced FDP is free of interference throughout dynamic occlusion. This method is susceptible to errors due to the complexity and individuality of the masticatory system. These errors may result in occlusal discomfort, temporomandibular disorders, or the failure of the inserted FDP (Preis et al., 2014; Schnitzhofer et al., 2023). In the dental domain, functional analysis refers to a comprehensive examination and assessment of the dynamic interactions among the teeth, jaw joints, muscles, and other oral structures during various activities like biting, chewing, and speaking. Digital methods of recording the jaw movements are available (Revilla-León et al., 2023) but costly and therefore rarely employed. Consequently, insertion of FDPs often requires adaptation and reworking. These steps result in elevated treatment cost and duration alongside a reduction in overall stability due to the partial weakening of the material. Adverse effects include surface damage, increased roughness, and the occurrence of fractures within the FDP.

### 1.1 State of the art of digital dental restorations

Various commercial software applications are available for designing FDPs such as CEREC by Dentsply Sirona or CARES^®^ by Straumann^®^ as well as ongoing research employing non data-driven conventional reconstruction techniques (Blanz et al., 2004; Zheng et al., 2011; Jiang et al., 2016). For example, Blanz et al. (2004) utilized the Bayesian maximum posterior probability in a non-iterative process to calculate an optimal reconstruction regarding fitting quality and plausibility. In contrast, Jiang et al. (2016) employed an iterative deformation approach in which salient features were identified by applying Morse theory (Matsumoto, 2002) and a standard tooth was modulated through iterative Laplacian surface editing (Sorkine et al., 2004) and mesh stitching. The proposed method is versatile and applicable to a range of tooth types including incisors, canines, premolars, and molars. Zheng et al. (2011) proposed a similar method involving morphing a standard tooth by extracting the contours of the cavities allowing a set of feature points to be matched on an available tooth preparation.

In light of the still existing problems in the quality of reconstruction, a data-driven approach based on deep learning can be pursued. Although deep learning has found broad applications in segmentation and classification within dentistry (Le Son et al., 2018; Wu et al., 2018; Lai et al., 2021; Rajee and Mythili, 2021), the use of generative network architectures for the generation and reconstruction of human teeth has been explored by only a few research groups. Derived from a 3D mesh object as the primary input format, the standard transformation of the raw data typically entails converting the data into one of two formats. The widespread availability and accessibility of 2D generative network architectures tailored for image inference (Isola et al., 2017; Karras et al., 2017, 2020a,b; Pandey et al., 2020; Ding et al., 2021; Lei et al., 2022; Tian et al., 2022c) have prompted the adoption of a 2D depth map representation for the data in various studies (Hwang et al., 2018; Yuan et al., 2020; Tian et al., 2021, 2022a,b).

Hwang et al. (2018), Yuan et al. (2020), and Lee et al. (2021) addressed the tooth reconstruction problem using a conditional GAN based on the pix2pix (Isola et al., 2017) architecture. In this model, the encoder learns a latent representation of the input data, which is then used to directly generate the full tooth reconstruction.

Tian et al. (2021) extended their previously proposed model (Yuan et al., 2020) in order to develop a dental inlay restoration framework for a total of 5 different inlay types. It employs a pix2pix (Isola et al., 2017) generator with a wasserstein loss (Yang et al., 2018), two discriminators, and a groove parsing network. The network is trained on very specific preparation types and its performance with other preparation types has not yet been validated.

Tian et al. (2022a) used a similar approach for the reconstruction of full occlusal surfaces with a dilated convolutional-based generative model and a dual global-local discriminative model. The proposed generative model utilizes dilated convolution layers to generate a feature representation that preserves the clear tissue structure, while the dual discriminative model employs two discriminators to jointly assess the input. The local discriminator only focuses on the defective teeth to ensure the local consistency of the output. The global discriminator evaluates whether the generated dental crown is coherent as a whole by examining the missing and adjacent teeth.

In their latest article, Tian et al. (2022b) proposed a network that employs a two-stage GAN process. In the first stage, the network generates the basic shape of the occlusal surface that satisfies the spatial positional relationship between the prepared jaw, opposing jaw, tooth type label, and gap distance between the two jaws. In the second stage, the network refines the details of the occlusal surface by incorporating information about the fossa and occlusal fingerprint.

While the previously mentioned studies used 2D representations of the 3D data for the implementation of the restoration methods, 3D data can be directly utilized. The suitability of the 2D depth map representation is restricted by the intrinsic characteristics of the data, limiting its suitability to a specific maximum quantity of teeth in the input data. More precisely, studies utilizing 2D data representations have concentrated on reconstructing the occlusal surface of a single tooth, with a maximum of three teeth per input image. The targeted tooth preparations for restoration were accompanied by two adjacent teeth in the same depth map, along with three antagonists and the gap distance between the two jaws in terminal occlusion (Hwang et al., 2018; Yuan et al., 2020; Tian et al., 2021), each saved as a separate image. Moreover, Tian et al. (2022a,b) broadened the scope of input data by integrating extracted dental biological morphology, including features such as the occlusal groove and the occlusal fingerprint (Kullmer et al., 2009). The ground truth in these studies consisted of single crowns designed by a technician.

When dealing with input data that involves a higher quantity, type, or combination of teeth, such as an entire upper or lower jaw, the practicality of the 2D representation diminishes due to data loss. This loss is primarily attributed to undercuts in the data that cannot be adequately captured through linear projection methods. As a result, incorporating a 3D representation proves beneficial. Zhu et al. (2022) and Feng et al. (2023) advocated for the utilization of transformer networks in combination with dynamic graph convolutional neural networks (Wang et al., 2018). The input data was represented as a point cloud. Chau et al. (2023) and Ding et al. (2023) opted for a 3D-GAN architecture (Wu et al., 2016). While the exact data format was not specified, voxelized points are assumed to be utilized due to the employed architecture (Wu et al., 2016). The selection of teeth for reconstruction varies, ranging from an arbitrary tooth (Zhu et al., 2022) to specific molars (Chau et al., 2023; Ding et al., 2023) or incisors (Feng et al., 2023). Unlike 2D methods, the input data is no longer confined to the occlusal surfaces of a limited number of teeth. Instead, it allows for the inclusion of the entire upper or lower jaw (Zhu et al., 2022; Chau et al., 2023). As of now, no study has employed the complete upper and lower jaw as input data, resulting in information containment limited to the respective jaw.

### 1.2 Summary

In this article, a data-driven approach for the partial reconstruction of occlusal surfaces is proposed. The use of a GAN (Goodfellow et al., 2014) is pursued. Since the stability of the training process of immature network architectures remains an ongoing problem, StyleGAN-2 (Karras et al., 2020b) is used as the main network for generating the occlusal surfaces. It is a broadly available and maintained state of the art architecture that shows great capabilities in terms of output quality and stability of the training process. To integrate the data set that comprises 92 3D mesh files of single molar crowns with the StyleGAN architecture, a normalized 2D projection method is proposed. The trained network's reconstruction abilities are then demonstrated for 4 common inlay types using a Bayesian Image Reconstruction method (Marinescu et al., 2020). For this purpose, the data is pre-processed to extract the necessary information of the tooth preparations required for the reconstruction and the initial loss is modified. Finally, the superior quality of the GAN-based restorations against a clinical procedure for CAD inlay fabrication is demonstrated. The results indicate the effectiveness of the proposed approach for the partial reconstruction of occlusal surfaces.

Differing from the aforementioned approaches, our method decouples individual components of the process, such as the 2D-projection, teeth generation, and reconstruction. This separation aims to simplify the application of further enhancements to each component. In the event of external developments, such as a new StyleGAN release, the modular nature of our approach allows for updates to maintain state-of-the-art performance.

In contrast to the inlay restoration network proposed by Tian et al. (2021), our method diverges by not being initially trained as a completion network. Instead, it functions as a generative network without a predefined target. The downstream optimization-based reconstruction method is not part of the training process, enabling the use of arbitrary restoration types. The employed GAN is thus capable of handling a diverse range of inlay geometries without necessitating time-consuming retraining.

Both approaches share a common foundation in the initial transformation of the tooth based on its bounding box. However, in the method proposed by Tian et al. (2021), the optimization of the spatial information contained in the depth map is achieved through the optimization of the projection parameters with respect to the resulting image's entropy. In our approach, we leverage the principle components analysis (PCA) of the occlusal surface to identify an optimal projection plane that maximizes the spatial information in the projection.

Moreover, unlike the data quantity integrated by the previously discussed methods (Hwang et al., 2018; Yuan et al., 2020; Tian et al., 2021, 2022a,b), our proposed method utilizes a dataset comprising 92 mesh files of single molar crowns. This showcases the proficiency of the 2D methodology in restoring varying extents of the occlusal surface with a minimal amount of data.

## 2 Materials and methods

The reconstruction of the occlusal surfaces is achieved by means of a 2 step process. This process includes a generating component with the basic knowledge of the morphology of teeth and a reconstructing component that uses the prior for the creation of unknown partial occlusal surfaces based on the remaining tooth data.

In the first step, the StyleGAN-2 (Karras et al., 2020a,b) network is trained using the available data. This enables the ability to generate a wide variety of occlusal surfaces. Adam (Kingma and Ba, 2014) is used as the optimization algorithm for both, the generator and discriminator network with α = 0.002, β_1_ = 0.0 and β_2_ = 0.99 without any learning rate decay or ramp down (Karras et al., 2017). The minibatch size for the given resolution is set to 32, the augmentation (Karras et al., 2020a) is disabled and the weight for the path length regularization term γ_*pl*_ is set to 10 with a decay coefficient of β_pl_ = 0.99. The training process concludes once 7 × 10^5^ images have been shown to the discriminator. A comprehensive description of the style-based GAN architecture is presented by Karras et al. (2017, 2020b). The data is split into a training and test data set that comprise 89 and 3 samples respectively. The training data is mirrored with respect to the vertical axes of the images yielding a total of 178 training images. The optimizer parameters are determined according to the recommendations of the original authors (Karras et al., 2017), while the remaining hyperparameters are selected to optimize the reconstruction performance of the trained generator.

In a second step, the partial reconstruction of the teeth is handled by a downstream optimization process that is derived from the Bayesian Image Reconstruction method as proposed by Marinescu et al. (2020). Here, the fixed generator *G*(***w***) of the fully trained StyleGAN-2 network serves as the underlying model for the optimization process. The input latent vector ***w*** is used to control the features of the generated occlusal surfaces ***x***_synth_ = *G*(***w***). The process employs an Adam (Kingma and Ba, 2014) optimizer with α = 0.05, β_1_ = 0.9 and β_2_ = 0.999.

In order to apply the reconstruction method, a complete image with size *w* × *h* and a dedicated binary mask ***M*** of the same size are required. During the reconstruction process, this mask is applied to both the true and the synthesized images ***x***_synth_ according to


(1) $$\begin{array}{l} x_{\text{cor}}=f_{\text{cor}}\left(x_{\text{cln}}\right)=x_{\text{cln}}⊙M. \end{array}$$

The masking operation *f*_cor_ is characterized by the Hadamard product of the binary mask ***M*** ∈ {0, 1}^*w*×*h*^ and the desired clean image $x_{\text{cln}}=G\left(w^{*}\right)\in {\mathbb{R}}^{w\times h}$. The Bayesian maximum a posteriori estimate over the latent vector $w\in {\mathbb{R}}^{512\times n_{\text{res}}}$ is depicted as w^*^ (Marinescu et al., 2020).

The StyleGAN proprietary parameter *n*_res_ is dependent on the resolution of the used images and can be calculated with Equation 2.


(2) $$\begin{array}{l} n_{\text{res}}=2\left(\log_{2}\left(w\right)-1\right)\forall w=h\wedge \log_{2}\left(w\right)\in {\mathbb{N}}_{\neq 0}. \end{array}$$

The solution to the optimization problem $x_{\text{cln}}^{*}$ can then be merged with the masked image according to Equation 3.


(3) $$\begin{array}{l} x_{\text{merge}}=x_{\text{cor}}+{\left(M-𝟙\right)}^{\text{abs}}⊙x_{\text{cln}}^{*}. \end{array}$$

The (·)^abs^ operator represents a copy of the argument matrix with all element-wise absolute values and 𝟙 denotes an all-ones matrix of the same size as ***M***.

### 2.1 Creation of a 2D tooth data set

In order to utilize the StyleGAN network architecture, the 3D mesh data needs to be projected into the 2D image space $X\in {\mathbb{R}}^{w\times h}$ in a normalized and repeatable manner. This involves normalizing the 3D orientation of the object followed by a 2D projection.

#### 2.1.1 Normalization of the 3D orientation

The normalization process consists of 2 essential steps. The first step aims to achieve independence of subsequent processing steps from the underlying intrinsic coordinate systems, which may vary due to changes in the used 3D data retrieval method. This enables identical teeth with different coordinate systems to produce identical results. A second step focuses on improving the orientation of the occlusal surface to ensure optimal data retention.

To achieve independence of the underlying coordinate system, the orientation of the body's bounding box is utilized. This box is defined as a cuboid with a minimum volume to encompass all points of the associated body. The orientation of the bounding box is determined by the rotation matrix ***R***_bb_ with respect to the initial coordinate system. The tooth, represented by the points ***P***_0_, can then be transformed using the inverse rotation matrix according to


(4) $$\begin{array}{l} P=R_{\text{bb}}^{-1}P_{0}. \end{array}$$

**Figure 1:**
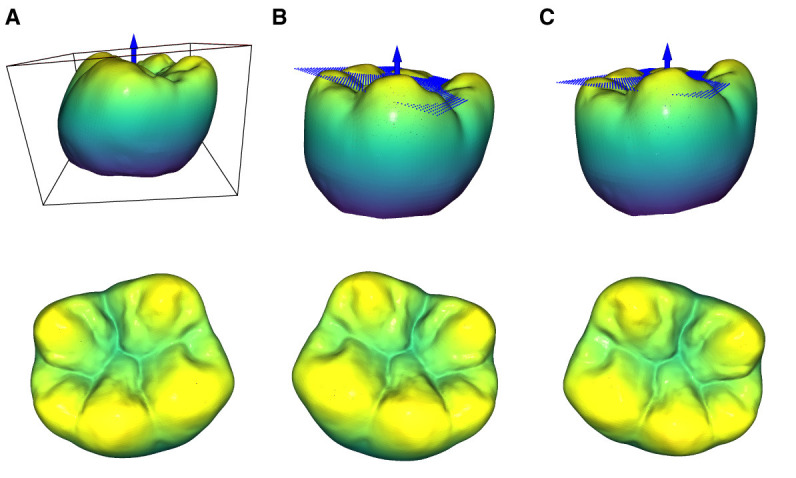
Representation of the 3 orientation stages of a tooth during the normalization process. The upper row shows the views with the bounding box and the planes that are defined by a subset of the occlusal surface points according to Equation 7. **(A)** Initial orientation of the tooth with the associated bounding box. It can be seen that the bounding box is not aligned with the coordinate system. **(B)** Orientation after the bounding box transformation according to Equation 4. **(C)** Orientation after the subsequent plane rotation according to Equation 8.

Figures 1A, B illustrate the top view of the tooth prior to and after the transformation in Equation 4. At this point, the transformed tooth's top view does not uniformly depict the occlusal surface as it lacks proper rotation around the *x* and *y* axes. The *z*-axis is represented by a blue arrow for reference.

Therefore, the second step is needed to increase the number of points that define the occlusal surface in the top view of the tooth by means of a second transformation. Due to the complex geometry of the occlusal surface, its orientation is approximated by an optimal fitting plane


(5) $$\begin{array}{l} \begin{array}{c} ax_{i}+by_{i}+cz_{i}=d,\\ n^{\text{T}}p_{i}=d. \end{array} \end{array}$$

The parameters of the plane can be obtained by performing a principal component analysis on a subset of points ***P*** that characterize the occlusal surface (Jacquelin, 2011). The 2 eigenvectors of the covariance matrix ***K***_***P***_*i*_***P***_*i*__ with the largest associated eigenvalues ***v***_1_ and ***v***_2_ define the orientation of the plane passing through the mean value of the point set **μ**_***P***_. Therefore, each point on the plane can be calculated according to


(6) $$\begin{array}{l} p_{i}=\mu_{P}+r_{1}v_{1}+r_{2}v_{2}, \end{array}$$

where *r*_*i*_ ∈ ℝ denotes an arbitrary real number and the normal vector of the plane ***n*** according to Equation 5 is represented by the eigenvector with the smallest associated eigenvalue ***v***_3_. Finally, substituting Equation 6 into Equation 5 with ***n*** = ***v***_3_ yields the final equation of the plane


(7) $$\begin{array}{l} v_{3}^{\text{T}}p_{i}=v_{3}^{\text{T}}\mu_{P}. \end{array}$$

The inverse of the plane's rotation matrix can now be used to perform a rotation of the tooth with respect to the initial *xy*-plane while leaving the *z* rotation constant according to


(8) $$\begin{array}{l} P=R_{\text{P}}^{-1}P_{0}. \end{array}$$

The rotation matrix is calculated based on the orientation of the plane with Equation 9.


(9) $$\begin{array}{l} \begin{array}{c} R_{\text{P}}=\left[\frac{x_{\text{F}}}{||x_{\text{F}}|{|}_{2}},\frac{y_{\text{F}}}{||y_{\text{F}}|{|}_{2}},\frac{z_{\text{F}}}{||z_{\text{F}}|{|}_{2}}\right],\\ x_{\text{F}}={\left[1,0,z_{\text{P}}\left(1,0\right)-z_{\text{P}}\left(0,0\right)\;\right]}^{\text{T}},\\ y_{\text{F}}={\left[0,1,z_{\text{P}}\left(0,1\right)-z_{\text{P}}\left(0,0\right)\;\right]}^{\text{T}},\\ z_{\text{F}}=x_{\text{F}}\times y_{\text{F}}. \end{array} \end{array}$$

By solving Equation 5 for *z*, the function *z*_P_(*x, y*) can be obtained. The resulting plane and the subsequent transformation can be seen in Figures 1B, C.

**Figure 2:**
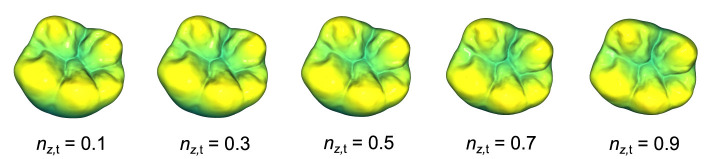
Representation of the top view of an exemplary tooth after a rotation with different threshold values *n*_*z*,t_ for the retrieval of a plane using PCA. The orientation of the calculated plane determines the applied rotation.

As already mentioned, the plane to describe the orientation of the occlusal surface is calculated based on a subset of points to reduce noise and improve the final position of the tooth. After the bounding-box dependent pre-rotation of the tooth according to Equation 4, the intrinsic normal vectors of the occlusal surface point in the positive *z*-direction. Therefore, their respective *z* components can be used to obtain the point subset ***P***(*n*_*z,i*_ > *n*_*z*,t_) for the calculation of the plane. Figure 2 depicts the top view of the resulting rotated teeth for different threshold values ranging from 0.1 to 0.9 with *n*_*z*,t_ = 0.9 yielding the best visual results. Namely, the respective point subsets show that the points assigned to the maxima of the cusps as well as the minima of the fossa are significant for determining a plane that accurately captures the overall orientation of the occlusal surface.

#### 2.1.2 Dimensionality reduction

The next step involves projecting the normalized 3D model of the tooth into a 2D image space to generate a depth map of the occlusal surface. The projection plane is positioned parallel to the *xy* plane of the normalized model. The final projections have a resolution of 256 × 256 with a bit depth of 8 bits, which allows for possible values ranging from 0 to 255 for every pixel. The selected resolution shows a good compromise between the duration of the training process and a sufficiently accurate representation of the data. The size of the quadratic projection plane in mm is determined by the maximal expansion in the *x* and *y* direction of the entire data set with an additional positive offset to ensure that the outer contour of the largest tooth does not touch the borders of the image. To normalize the position of the tooth, it is centered inside the projection plane. Additionally, the *z* position of every point is normalized using Equation 10.


(10) $$\begin{array}{l} p_{i}=p_{i,0}+{\left[0,0,z_{\text{norm}}-z_{\text{max}}\right]}^{\text{T}}\forall i\in \left[1,n\right]. \end{array}$$

The global parameter *z*_norm_ is fixed for the whole data set. The maximum *z* direction for the respective tooth is represented by *z*_max_. To achieve maximal spatial resolution, the position of the projection plane is determined by the new value of *z*_max_ = *z*_norm_. The projection of each individual pixel on the grid is then executed according to Equation 11.


(11) $$\begin{array}{l} x_{i,j}=\{\begin{array}{ll} \frac{255}{z_{\text{norm}}}\underset{z}{\max}z_{i,j} & \exists \;z_{i,j}>0\\ 0 & \left(∄\;z_{i,j}\in \mathbb{R})\vee (z_{i,j}\leq 0\right) \end{array},\\ \;\forall \;i,j\in [0,255] \end{array}$$

**Figure 3:**
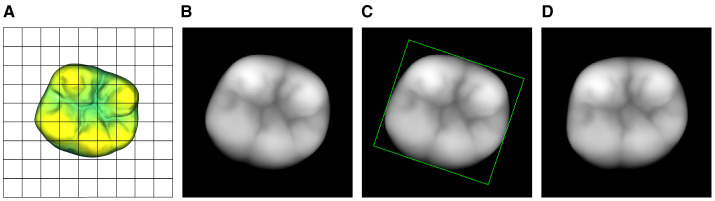
Visualization of the 2D projection process including the subsequent normalization of the tooth's orientation inside the depth map. **(A)** View of the normalized 3D model through the projection plane. **(B)** 2D projection of **(A)**. **(C)** Bounding rectangle of the tooth's depth map. **(D)** Rotated tooth with a bounding rectangle that is parallel to the edges of the images.

A single pixel is represented by *x*_*i,j*_ and the corresponding *xy*-coordinates of the image are represented by *x*_*i*_, *y*_*i*_. The grid-coordinate dependent distance *z*(*x*_*i*_, *y*_*j*_) is depicted as *z*_*i,j*_. Namely, this process captures the surface of a given object while neglecting all points with a vertical distance greater than *z*_norm_ from the object's highest point. Figures 3A, B showcase the view of the 3D model through the projection plane along with the final 2D depth map of the tooth in the 8-bit grayscale image space $X\in {\mathbb{R}}^{w\times h}$.

To further improve the normalized depth map representation of the occlusal surface, a rotation around the *z* axis is performed in the 2D space. The *z* axis is perpendicular to the image plane. Similar to the described 3D bounding box as a reference for the orientation of the volumetric model, a bounding rectangle is used to determine the orientation and position of the 2D projection as shown in Figure 3C. The 2D projection of the tooth with *x*_*i,j*_ ≠ 0 ∀ *i, j* ∈ [0, 255] can then be transformed such that the position of the rectangle's center coincides with the center of the image. Additionally, the *z* rotation is adjusted such that the bounding rectangle is parallel to the edges of the image and the buccal aspects of the tooth are located in the bottom half of the image. The final 2D projection of the 3D tooth is shown in Figure 3D.

The described method requires the available mesh data to be converted to a 3D point cloud. Based on empirical testing, the number of sampled points is set to 3.3 × 10^6^ for the given resolution. Due to the non-deterministic nature of the executed sampling process, the 95% CI RMSE of the 3D to 2D projection is analyzed. This involves projecting every tooth from the entire data set *n* = 100 times into the 2D space and comparing each projection to its corresponding reference image by calculating the RMSE according to Equation 12.


(12) $$\begin{array}{l} {\text{RMSE}}_{\text{mm},i}=c_{\text{pix}\to \text{mm}}\text{RMSE}\left(x_{0},x_{i}\right)\forall i\in \left[1,n\right]. \end{array}$$

The fixed factor *c*_pix→ mm_ is used for the conversion from an 8 bit unsigned integer to a float with its physical unit mm. The result of this procedure is a total RMSE of [3.81, 3.97] × 10^−3^ mm (including background), which translates to a difference per non-zero pixel of [6.57, 6.68] × 10^−3^ mm (excluding background). Therefore, a deviation of this magnitude can be neglected in further proceedings as it represents the quantitative difference between two images that originate from the same 3D model with visually indistinguishable projections.

### 2.2 Extraction of the 2D preparation area

**Figure 4:**
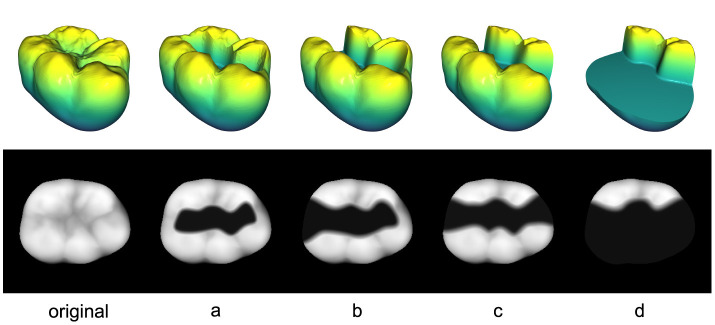
3D and 2D representations of all 4 tooth preparations (a–d) including the original tooth on the left.

For the forthcoming experiments, the teeth in the 3D test data set are prepared by a dental professional to receive 4 distinct types of commonly used inlays as illustrated in Figure 4. These inlays are denoted with letters from “a” to “d” with the specific order determined by the size of the inlay to be received. Since the proposed method necessitates 2D data, the tooth preparations are projected into the 2D image space utilizing the techniques described in Section 2.1.2. This yields the images a-d displayed in the second row of Figure 4 for an exemplary tooth.

To apply the proposed reconstruction method, the prepared area of the tooth model has to be extracted as a 2D mask ***M***, which allows for the corruption process *f*_cor_ to be performed as described in Equation 1. One possible approach is the generation of a mask based on the 3D edges that were created as a result of the tooth preparation as shown in Figure 4, where the breaks in continuity on the occlusal surfaces of the prepared teeth are clearly visible. The boundaries of the masks must be coincident with the exact edge lines of the prepared teeth for the final merging process. Otherwise, the resulting reconstructed inlay will not satisfy the boundary condition


(13) $$\begin{array}{l} p_{\text{recon},i}=p_{\text{prep},i}\left\{\forall i\in \left[1,n\right]|p_{\text{prep},i}=p_{\text{edge},i}\right\} \end{array}$$

for all points on the preparation edge.

In order to define the mask, all points that are associated with the tooth preparation process, i.e., the points located inside and below the preparation edge, have to be extracted in the 3D space. It is assumed that the normal vectors ***n***_*i*_ for each triangle in the mesh are perpendicular to the gradients according to Equation 14.


(14) $$\begin{array}{l} n_{i}⊥\nabla P\left(x_{i},y_{i},z_{i}\right). \end{array}$$

**Figure 5:**
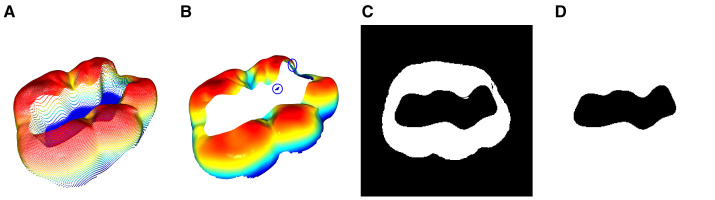
Depiction of the 3D mask area extraction process: **(A)** Pointcloud of the 3D tooth preparation as shown in Figure 4. **(B)** Pointcloud after a crop in *z*-direction in order to remove the bottom part of the the preparation area and the extraction of all vertices with normals ***n***_*i*_ that satisfy the condition *n*_*z,i*_ > *n*_*z*,t_. It can be seen that small residual point accumulations (blue circle) associated with the preparation area are still present. **(C)** The projection of **(B)** into the 2 dimensional binary space where every pixel with a non zero value is set to 255 according to Equation 16. **(D)** The final 2D binary mask ***M*** after manual post processing of **(C)** to fill the gaps that result from the aforementioned artifacts.

The 3D point cloud is represented by ***P***. Therefore, the *z*-component *n*_*z,i*_ of the normal vector is utilized to eliminate the points related to the preparation area. As shown in Figure 5A, these points exhibit two distinct characteristics. The bottom part is below a specific *z* threshold, which can be easily cropped, and the lateral area is much steeper than the occlusal surface of the tooth. Therefore, the heuristic in Equation 15 is sufficient to produce satisfactory results, as illustrated in Figure 5B.


(15) $$\begin{array}{l} P_{\text{mask}}=P\left(n_{z,i}>n_{z,\text{t}}\wedge z_{i}>z_{\text{t}}\right). \end{array}$$

The thresholds *n*_*z*,t_ and *z*_t_ are individual, empirical values for each preparation. Residual artifacts, such as small point accumulations associated with the preparation area or tiny holes in the occlusal surface are not present in the direct vicinity of the preparation edge. Therefore, they do not affect the accuracy of the resulting mask as they do not interrupt the edge.

Figure 5C shows the 2D projection of the cropped point cloud in a binary space


(16) $$\begin{array}{l} x_{\text{bin},i,j}=\{\begin{array}{cc} 255 & x_{i,j}\neq 0\\ 0 & x_{i,j}=0 \end{array}\forall i,j\in \left[0,255\right]. \end{array}$$

The artifacts can now be manually corrected to create an enclosed binary mask ***M*** with a white background, as depicted in Figure 5D.

### 2.3 Introduction of a boundary loss term

A final preliminary step necessary for a smooth transition between the reconstructed and the real part of the combined image ***x***_merge_ is the extension of the initial reconstruction loss $L_{\text{init}}$ according to Marinescu et al. (2020). This is achieved by adding a term to the loss function that inspects pixels neighboring the edge of the black mask area *M*_*i*_ = 0 to ensure that the boundary condition (Equation 13) is met. Specifically, the loss increases with a larger deviation of the boundary pixels. The computation of the newly introduced term is expressed by Equation 17.


(17) $$\begin{array}{l} L_{\text{pixel,B}}=||f_{\text{cor,B}}\circ x-f_{\text{cor,B}}\circ G\left(w\right)|{|}_{2}^{2}. \end{array}$$

**Figure 6:**
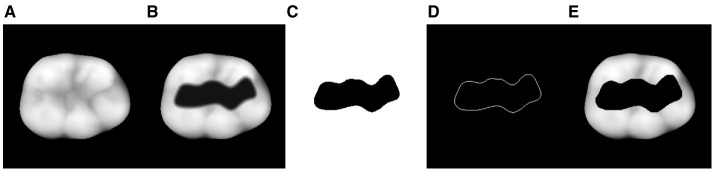
Development of the tooth image data from left to right: **(A)** Complete tooth ***x***_true_ (ground truth). **(B)** 2D projection of the 3D tooth preparation. **(C)** Extracted binary mask ***M*** that perfectly covers the prepared area. **(D)** Outer boundary ***M***_B_ of the binary mask ***M*** for the extended pixel boundary loss $L_{\text{pixel,B}}$. **(E)** 2D projection of the 3D tooth preparation with mask ***x***_cor_.

The symbol ||·||_2_ represents the *l*_2_ norm and the masking operation is denoted as *f*_cor,B_ with the boundary mask ***M***_B_, as shown in Figure 6D. The new cost function can be calculated according to Equation 18.


(18) $$\begin{array}{l} L_{\text{B}}=L_{\text{init}}+\lambda_{\text{pixel,B}}L_{\text{pixel,B}}. \end{array}$$

The weight of the introduced loss term is represented by λ_pixel,B_. The weights of the initial loss term $L_{\text{init}}$ according to Marinescu et al. (2020) and the boundary loss term are set to λ_w_= -3.2, λ_colin_= 8.6 × 10^−4^, λ_pixel_= 6.7 × 10^−4^, λ_percept_= 9.2 × 10^3^ and λ_pixel,B_= 4.0 × 10^−4^.

## 3 Results

In the following section, the performance of the reconstruction method is presented using the test data set and all 4 types of preparations as shown in Figure 4. Furthermore, a full set of preparations for an exemplary tooth will be compared against the restoration results of a clinical procedure for CAD inlay fabrication.

### 3.1 Quantitative evaluation of the reconstructions

The images required for the upcoming reconstruction and evaluation process are shown in Figure 6. Figures 6B–D are explicitly utilized for the reconstruction process, while Figure 6A is the ground truth ***x***_true_ for the quantitative evaluation of the output.

With the used NVidia GTX 1080 ti, the reconstruction process takes about 120 s with 16 iterations per second and a maximum of *n*_R_= 2 × 10^3^ optimization steps. The stagnation of change of the reconstruction error occurs at a calculated point of diminishing returns *n*_dr_ = [201, 212] for all image mask combinations. For performance oriented applications, the optimization process could thus be executed in about 12 s instead of 120 s.

For the following evaluation and comparison, the reconstruction with the minimal loss $L_{\text{B}}$ after *n*_R_= 2 × 10^3^ optimization steps is used for each run. Since all reconstructions are based on a known ground truth ***x***_true_ of the complete tooth, the RMSE between the merged reconstruction and the ground truth is used as the comparison metric. The calculation of the RMSE in mm is performed including the background of the images.

**Figure 7:**
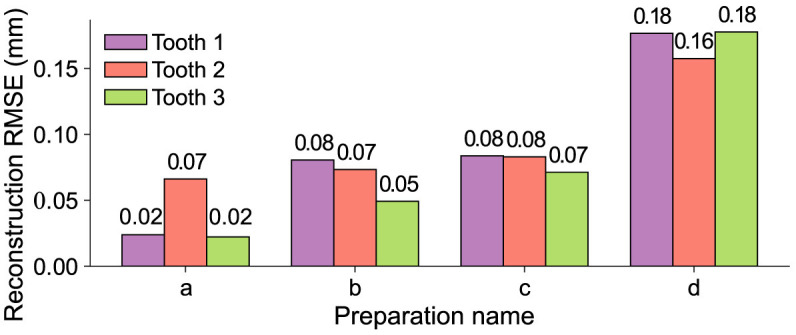
RMSEs of the merged reconstructions ***x***_merge_ and the respective ground truth ***x***_true_ for all teeth from the test data set. The development of the error across the different masks shows a steady increase with respect to the size of the removed area.

The resulting values for all merged reconstructions ***x***_merge_ and their respective ground truth ***x***_true_ for the test data set are shown in Figure 7. To facilitate a better assessment of the displayed data, it should be considered that the RMSE of the 3D → 2D projection is equal to [3.81, 3.97] × 10^−3^ mm for identical input data. Therefore, a RMSE of this magnitude represents a perfect reconstruction. It can be seen that across the entire data set, there is a steady increase in the RMSE as the size of the removed area increases. In terms of quantitative reconstruction quality, the best reconstructions for preparation “a” exhibit an error that deviates from a theoretically perfect reconstruction by a factor of 5.

**Figure 8:**
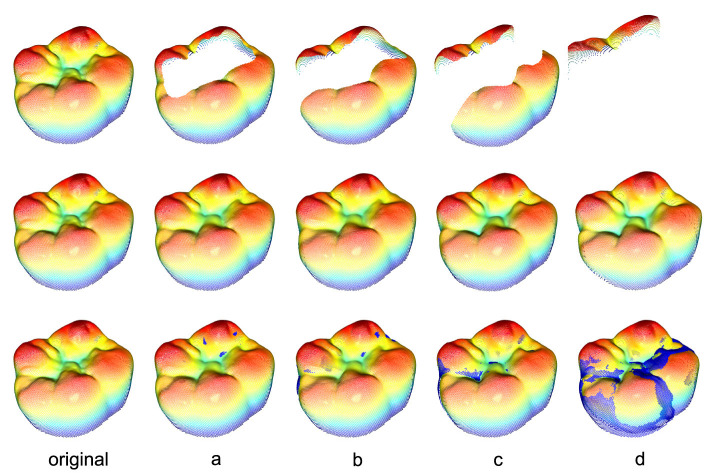
Reconstructions of all 4 tooth preparations for an exemplary tooth including the ground truth in the first column. The second row displays the mere reconstructions whereas the third row shows the reconstructions including all true points (blue) with an error-distance greater than 0.1 mm. The exact distribution of all distances for every preparation can be seen in Figure 9.

To gain a better understanding of the relationship between the quantitative RMSE and the quality of the reconstruction, the visual appearance of the tooth has to be considered. Figure 8 shows the masked tooth preparations in the first row, the associated reconstructions in the second row and the visual difference between the reconstruction and the ground truth in the last row. The visual difference is calculated by including all points of the original tooth with an error-distance greater than 0.1 mm.

**Figure 9:**
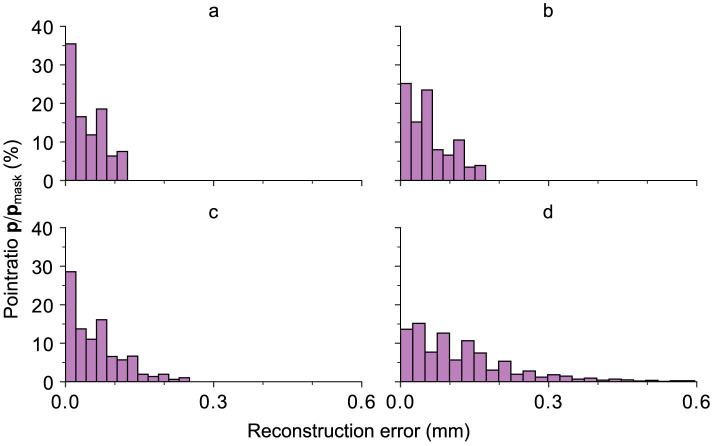
All relative ratios of the points with a certain distance, depicted on the abscissa, with respect to the total number of points of the black mask area (*M*_*i*_ = 0) for every preparation within the 95th percentile for an exemplary tooth. The results are related to the images displayed in Figure 8. As expected, an increase in the removed area yields an overall worse distribution of the points within this area. However, as already shown in Figure 8, most of the reconstructed area for preparations a–c is located at an error distance <0.1 mm and <0.2 mm for preparation “d”.

Figure 9 depicts the relative distribution of the absolute error-distances of the reconstruction with respect to the total number of points inside the black mask area (*M*_*i*_ = 0) for every preparation within the 95th percentile distance for the same tooth. The 95th percentile distance is utilized as a practical threshold for focusing on the majority of data points while excluding the extreme values. The highest 5% of error distances do not contribute to the overall result for a continuous occlusal surface. This choice facilitates a more meaningful and representative visualization of the reconstruction outcomes. It can be seen that an increase in the removed area yields an overall worse distribution of the points within this area. Most of the reconstructed area for preparations a–c is located at an error distance <0.1 mm and <0.2 mm for preparation “d”.

### 3.2 Evaluation of the reconstruction method

In the following, the behavior of the proposed method with respect to linearly increasing mask sizes with a fixed geometry is evaluated. This involves masking the teeth from the test data set with a filled circle that removes 10% to 70% of the tooth area. The position and maximum relative size of the circle are determined by the biggest inscribing circle for each tooth. For comparison, the relative cut-out area of the available real preparations ranges from approximately 30% to 80% with the largest real mask exceeding the removed area of the largest circle. To ensure better comparability, the circles are kept inside the respective teeth and do not touch or cross the outer contour. This limits the upper relative bound of the study to the smallest value of the biggest inscribing circle for each tooth from the test data set.

**Figure 10:**
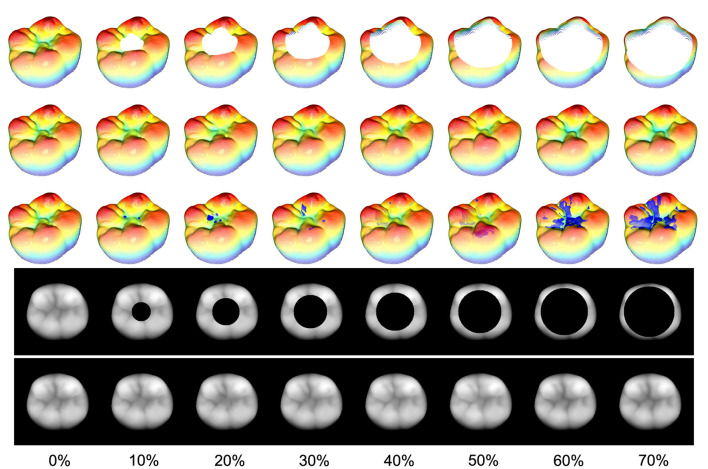
Rising area masks applied to an exemplary tooth with their associated reconstructions and the ground truth (first column). The relative removed area, related to the nonzero area of the 2D image, is located at a range from 10% to 70%. The third row of the 3D representations of the reconstructed teeth includes the true points (blue) with an error-distance greater than 0.1 mm. It can be seen that even for a 70% removal of the occlusal surface, the proposed method is able to reconstruct the missing part such that there are no major visible differences between the reconstruction and the ground truth.

Figure 10 illustrates the masks applied to an exemplary tooth along with the associated reconstructions, the ground truth as well as the 3D projections of the 2D images. The third row of the 3D projections contains representations of the reconstructed teeth including the true points (blue) with an error-distance greater than 0.1 mm.

**Figure 11:**
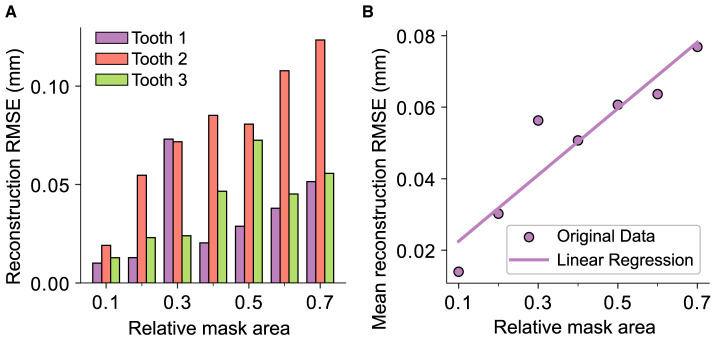
Representation of the reconstruction RMSE and the relative masked area for every tooth in the test data set, i.e., the area removed by the mask with respect to the whole area of the tooth where *x*_*i,j*_ ≠ 0. **(A)** The distribution across the whole test data set. **(B)** The correlation between the mask area and the RMSE represented by a linear regression over the mean values of the data displayed in **(A)** with *R*^2^ = 0.94 and *p* = 0.002.

The results coincide with the previously presented conclusions, as all the reconstructions in this study exhibit visually nearly indistinguishable quality assuming the real scale of a human tooth. Despite this, the spatial frequency within the reconstruction area experiences a decrease, even with the smallest mask applied. The overall quantitative results are presented in Figure 11. The bar plot illustrates the RMSE for each tooth, while the scatter plot shows the mean reconstruction RMSE across all teeth as a function of the relative mask area. Assuming that the masks do not intersect the outer contour of the tooth, it is evident that there exists a significant linear correlation between the reconstruction error and the relative area of the removed surface with an *R*^2^ of 0.94 and a *p*-value of 0.002. However, it should be emphasized that the reconstruction outcomes may differ for masks with the same relative size depending on their shape and position.

**Figure 12:**
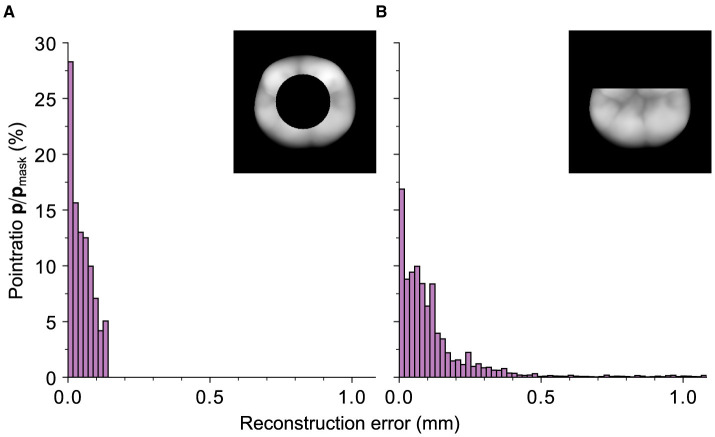
Representation of the relative ratios of the points with a certain distance, depicted on the abscissa, with respect to the total number of points of the black mask area (*M*_*i*_ = 0) within the 95th distance percentile for an exemplary tooth. **(A)** The values for a circle mask with a relative cut out area of 0.3 and a RMSE of 0.024 mm. **(B)** Mask with an equivalent cut-out area but a partial removal of the outer contour with a RMSE of 0.15 mm. It can be seen that the reconstruction with the latter mask performs worse although an equivalent number of pixels is removed.

The previous results indicate that the quality of the reconstruction can be influenced by the outer contour information of the tooth. Specifically, the reconstruction performs worse when a mask intersects the outer boundary. To investigate this behavior, a circle mask and a mask that removes an equivalent area of the tooth while also intersecting the upper outer contour are compared. Both masks remove 30% of the original tooth surface area. Figure 12 depicts the direct comparison of the error-distances for the respective reconstructions. The “boundary cut” reconstruction exhibits significantly higher error-distances due to the geometric uncertainty caused by the mask's intersection with the tooth's borders. With its RMSE of 0.15 mm, it is clearly outperformed by the reconstruction with a non-boundary cutting circle mask and a RMSE of 0.024 mm. These findings confirm the results from the real preparations.

### 3.3 Comparison with clinical restorations

**Figure 13:**
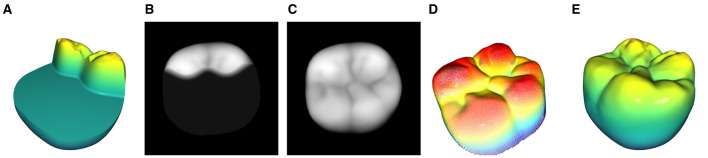
Complete process of the 3D mesh tooth preparation to the final merged mesh reconstruction. **(A)** The tooth is prepared by a dentist and saved as a mesh file. **(B)** 2D Projection of the preparation which serves as the base for the reconstruction process. **(C)** Fully reconstructed and merged 2D tooth representation **(D)** 3D projection of **(C)**. **(E)** Merged mesh with the inlay data from the reconstruction **(D)** and the base preparation **(A)**.

To validate the quality of the reconstructions with respect to its practical *in vitro* application, a domain-guided survey is conducted. In the course of this, the reconstructions for all preparations of an exemplary tooth are compared to the results of a clinical procedure for CAD inlay fabrication for the creation of inlay restorations. A dental technician is provided with the same preparations a-d as shown in Figure 4. These are processed with commercially available CAD/CAM software to generate the inlay restorations. On the other hand, Figure 13 illustrates the GAN-based reconstruction method from the 3D preparation in Figure 13A to the final merged 3D reconstruction in Figure 13E. The inlay is extracted from the final reconstruction using CAD software. The necessary tolerances for the joining process are applied by a technician.

**Figure 14:**
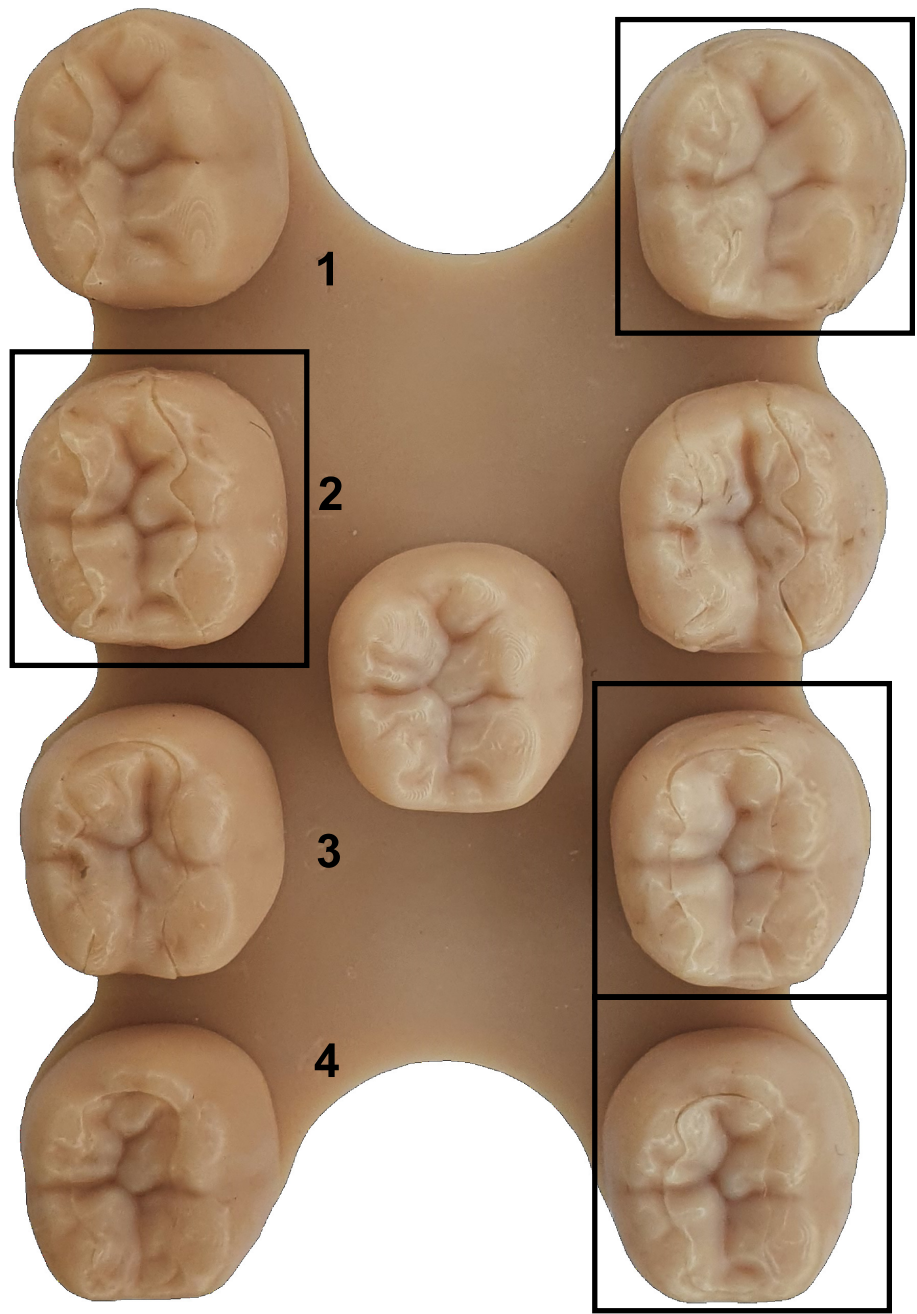
The base plate with all restorations for an exemplary tooth. It is divided into 2 full columns **(left, right)** with the restorations (rows 1–4) and a middle column that contains the ground truth. The marked reconstructions are based on the proposed method.

The baseplate with all preparations and the respective inlays are printed on a Formlabs Form 3b SLA printer using Formlabs Dental Model Resin. The base is divided into 2 columns (Figure 14 left, right) with the restorations (rows 1–4) and the ground truth in the center. To prevent bias toward one restoration type, the restorations are randomly shuffled between the 2 columns as shown in Figure 14.

The blinded survey is divided into 2 parts. In the first part, a group of dentists is asked to evaluate which restoration evokes a better overall visual impression, disregarding the joining operation. Following this, the ground truth is revealed and the visual quality is rated for all restorations on a five-step selection grid ranging from “0: very bad” to “4: very good” in comparison with the original tooth.

The results of the survey with a sample size of 6 are presented in Table 1. The findings indicate that the GAN-based restorations are preferred for all preparations except for “a” in the first part of the survey. However, when the restorations are compared with the ground truth, the GAN-based method consistently receives high ratings (“good”–“very good”). Particularly for the more complex restorations, the subjective quality of the GAN-based method surpasses the clinical procedure in both parts of the survey.

**Table 1:** Results of the clinical comparison with a sample size of 6.

**Prep. type**	**Favored GAN**	**GAN**	**Tech**.
a	17%	[2.5, 3.5]	[3.5, 4.0]
b	67%	[2.6, 3.8]	[1.0, 3.0]
c	100%	[2.6, 3.8]	[0.0, 0.7]
d	100%	[2.5, 3.5]	[1.3, 2.1]

Part 1: the second column shows the relative share of participants that favored the GAN reconstruction over the technician's reconstruction for every preparation type. Part 2: columns 2 and 3 show the 95% CI ratings for both restoration types (0–4 selection grid from “very bad” to “very good”). It can be seen that the GAN based restorations are favored for all preparation types except “a”.

## 4 Discussion

Based on the presented findings, it has been demonstrated that the StyleGAN-2 architecture is able to learn the correct representation of various occlusal surfaces using a limited 3D tooth data set. This allows for a downstream optimization process to successfully restore up to 80% of an unknown corrupted tooth. The data set used for training comprises 8-bit grayscale images $x_{\text{L}}\in {\mathbb{R}}^{256\times 256}$ with a black background. These images differ significantly in terms of detail, complexity, and resolution from the RGB images $x_{\text{RGB}}\in {\mathbb{R}}^{w\times h\times 3}$ typically used with the StyleGAN architecture. Despite the small size of the data set compared to popular data sets such as AFHQ CAT (5 × 10^3^ images, ℝ^512 × 512 × 3^) or CIFAR-10 (5 × 10^4^ images, ℝ^32 × 32 × 3^) (Karras et al., 2020a), the training process converges after 7 × 10^5^ images have been shown to the discriminator with good results in terms of quality and diversity of the generated teeth. The trained network is used in combination with the modified Bayesian Image Reconstruction method (Marinescu et al., 2020) to generate the occlusal surfaces for 4 common inlay restoration tasks. It is shown that the proposed method yields satisfactory visual and quantitative results for all preparations. Most of the points for the reconstructions of the preparations a–c are located at an error-distance <0.1 mm with RMSEs of 0.02 mm to 0.08 mm. The reconstructions for preparation “d” with a missing area of about 80% show RMSEs from 0.16 mm to 0.18 mm and a reconstruction error that is mostly located at distances <0.2 mm. As no additional information is available beyond the remainder of the prepared tooth, the results are considered to be very positive.

Even for the highly demanding preparation “d,” the reconstructed occlusal surfaces replicate the overall true appearance for every preparation. However, the method fails to adopt some of the high frequency spatial features in the mid region of the ground truth. A common issue for the open preparations b–d that cut the outer contour of the original tooth is the local shrinkage of the reconstruction near the outer boundaries of the ground truth. For masks that do not cut the outer contour of the target tooth, the proposed boundary loss $L_{\text{B}}$ implicitly solves the problem by ensuring that the pixels around the mask are assigned to the original values of the target. The trained model with its knowledge of the morphology of a real tooth now prohibits a pixel with a value of zero surrounded by non-zero pixels. This results in a watertight and smooth representation of the occlusal surface. For a mask that cuts the outer contour of the tooth, the boundary condition for the outer contour is *x*_*i,j*_ = 0, allowing the shrinkage of these regions near an outer boundary of the target. The most obvious display of this behavior is the distance representation of the reconstruction for preparation “d” in the lower right corner of Figure 8, which coincides with the respective distribution shown in Figure 9. The results in Figure 12 confirm these observations where 2 masks with identical relative corruption areas of 30% and different positions are compared. One of the masks intersects the outer boundary while the other mask is positioned in the center of the tooth.

A final comparison with a clinical procedure for CAD inlay fabrication demonstrates the overall superior quality of the GAN-based restorations. However, the conducted evaluation has some limitations. The sample size of 6 participants is relatively small and they may be biased toward the visual appearance due to the joining process. The 3D printed base plate with the restorations is fabricated with a model resin. This complicates the refining process of the joined restorations yielding worse visual results. In summary, the results indicate that the practical application of the proposed method is consistent with the quantitative results and can be considered successful.

Previous research for the data driven restoration of occlusal surfaces used encoder-decoder methods to directly generate the missing tooth data. Data sets ranging from teeth sectors to whole jaws were utilized in the 2D and 3D space. The employed networks have to be trained on data sets that are prepared for the specific restoration case. The proposed method achieves independence of the training of the network from the downstream optimization method. Therefore the GAN can be trained on data sets of full teeth, tooth sectors, or full jaws without specific preparation for the pursued reconstruction process. New data can be added to the data set with minimal effort.

Further improvements to the method can be achieved by incorporating information from the adjacent teeth. The network has to be trained with sectors of teeth or whole jaws and the opposing teeth or jaws have to be included in the optimization process. As the current data set consists of crown restorations, real teeth scan data should be utilized to ensure well fitting restorations for real world applications.

The current implementation of the StyleGAN architecture necessitates 2D representations of the input data. Consequently, the method's efficacy is confined to a specific maximum quantity of teeth within the input data. When confronted with datasets containing a greater quantity, variety, or combination of teeth, such as entire upper or lower jaws, the practicality of the 2D representation diminishes due to inherent data loss. This loss predominantly arises from undercuts in the data that cannot be adequately captured through linear projection methods. The use of 3D architectures offers a potential solution to alleviate these challenges. Recent studies exploring non-dental applications of 3D shape completion show promising results using diffusion-based architectures (Zeng et al., 2022), Transformers (Yu et al., 2023), or GANs (Sen et al., 2023). The application of these methods to the current problem could potentially yield more accurate and robust reconstructions.

## Data availability statement

The raw data supporting the conclusions of this article will be made available by the authors, without undue reservation.

## Author contributions

AB: Data curation, Formal analysis, Investigation, Methodology, Project administration, Software, Validation, Visualization, Writing—original draft, Writing—review & editing. MR: Conceptualization, Resources, Writing—review & editing. TS: Resources, Writing—review & editing. MG: Conceptualization, Project administration, Supervision, Writing—review & editing.
